# Photosynthesis and Respiration of Baltic Sea Benthic Diatoms to Changing Environmental Conditions and Growth Responses of Selected Species as Affected by an Adjacent Peatland (Hütelmoor)

**DOI:** 10.3389/fmicb.2019.01500

**Published:** 2019-07-04

**Authors:** Lara R. Prelle, Angelika Graiff, Sigrid Gründling-Pfaff, Veronika Sommer, Kana Kuriyama, Ulf Karsten

**Affiliations:** Institute of Biological Sciences, Applied Ecology and Phycology, University of Rostock, Rostock, Germany

**Keywords:** ecophysiology, microphytobenthos, heterotrophy, *Nitzschia dubiiformis*, *Actinocyclus octonarius*

## Abstract

Eight benthic diatom taxa (*Actinocyclus octonarius*, *Melosira moniliformis*, *Halamphora* sp. 1, *Halamphora* sp. 2, *Navicula perminuta*, *Navicula phyllepta*, *Nitzschia dubiiformis*, *Nitzschia pusilla*) were isolated from sediments sampled in the southern coastal brackish Baltic Sea and established as unialgal cultures. The coastal shallow water sampling area lies close to a fen peat site (Hütelmoor) and both are connected through an underground peat layer, which might facilitate organic matter and nutrient fluxes along the terrestrial-marine gradient. The photosynthetic performance of these diatoms was measured at different photon fluence rates (0–1200 μmol photons m^–2^ s^–1^, always recorded at 20°C) and different temperatures (5–40°C, always measured at saturating ∼270 μmol photons m^–2^ s^–1^), resulting in light saturation points between 32 and 151 μmol photons m^–2^ s^–1^ and maximum net primary production rates of 23–144 μmol O_2_ mg^–1^ Chl *a* h^–1^. None of the species showed severe photoinhibition, and hence all displayed a high photo-physiological plasticity. Photosynthetic oxygen evolution and respirational oxygen consumption between 5 and 40°C revealed eurythermal traits for half of the studied taxa as photosynthetic efficiency was at least 20% of the maximum values at the extreme temperatures. The remaining taxa also indicated eurythermal characteristics, however, photosynthetic efficiency of at least 20% was at a narrower temperature range [5 (10) °C to 30 (35) °C]. Species-specific optimum temperatures for photosynthesis (15–30°C) were always lower compared to respiration (25–40°C). *Actinocyclus octonarius* and *Nitzschia dubiiformis* were grown in different defined media, some enriched with Hütelmoor water to test for possible effects of organic components. Hütelmoor water media stimulated growth of both diatom species when kept in a light dark cycle. *Actinocyclus octonarius* particularly grew in darkness in Hütelmoor water media, pointing to heterotrophic capabilities. The benthic diatoms studied are characterized by high photo-physiological plasticity and a broad temperature tolerance to maintain high primary production rates under wide environmental fluctuations. Organic carbon fluxes from the Hütelmoor into the Baltic Sea may support mixo- and/or heterotrophic growth of microphytobenthic communities. These are essential traits for living in a highly dynamic and variable shallow water environment at the coastal zone of the Baltic Sea.

## Introduction

Microphytobenthic assemblages play an important ecological role in marine nearshore ecosystems as they are massively contributing to the marine primary production (Falkowski et al., 1998; Cahoon, 1999). The microphytobenthos is of high diversity as it can be composed of different taxa of euglenids, chlorophytes, cyanobacteria, dinoflagellates, and diatoms (Bacillariophyceae) (Colijn and De Jonge, 1984; Sundbäck and Miles, 2002; Launeau et al., 2018). Many studies, however, have shown that microphytobenthic communities in coastal regions are usually dominated by pennate, often epipelic motile diatom species, that move freely inside and on the sediment (Hillebrand and Sommer, 1997; Wasmund and Uhlig, 2003; Blommaert et al., 2018). As a result of their carbon fixation capacity, many of these pennate diatoms excrete sticky extracellular polymeric substances (EPS) leading to the formation of phototrophic biofilms on top of soft bottoms, thereby reinforcing the stability of sediments (Goto et al., 2001; De Brouwer et al., 2005; Beninger et al., 2019). In addition, EPS excretion facilitates motility of these protists, for example, through vertical movement into or out of the sediment as protective response to a range of environmental stressors (Cohn and Disparti, 1994; Shniukova and Zolotareva, 2015). Further important ecological functions include those as biological producers for oxygen and filter for other elemental fluxes (e.g., nutrients) at the sediment-water interface (Risgaard-Petersen et al., 1994), and as a major food source for benthic suspension- or deposit-feeders (Cahoon, 1999; Sackett et al., 2016). Benthic diatoms strongly benefit from the usually high nutrient concentrations in the pore water as a source of fuel for photosynthesis and growth (Admiraal, 1984), and they are involved in biochemical cycling of carbon, nitrogen, phosphorous, and silicate in shallow coastal waters (Morel and Price, 2003; Wilhelm et al., 2006).

Shallow coastal zones are quite sensitive to natural changes in abiotic factors due to their distinctive sea-land transition zone which is characterized by exchange processes between the terrestrial and marine compartments. This ecocline typically shows strong diurnal and seasonal fluctuations in meteorological conditions, causing increasing sea-land exchange processes, which might impact organisms living in this transition zone (Jurasinski et al., 2018). In a dynamic environment such as the coastal region of the Baltic Sea, light might be a limiting factor for benthic diatoms due to seasons, meteorological conditions, shading by phytoplankton blooms, turbidity and infauna sediment turbation. Consequently, photon fluence rates, for example, in the Wismar bight can even diurnally fluctuate between 389 and 2117 μmol photons m^–2^ s^–1^ (Woelfel et al., 2014). Nevertheless, the light climate in non-tidal coastal areas like the Baltic Sea seems to be sufficient for benthic diatoms to establish microphytobenthic biofilms (Miller et al., 1996). Depending on the sediment grain size, the thickness of these biofilms can vary as larger grains favor mainly motile diatoms to live between particles. Raphid diatoms vertically move in the sediment with a speed of 10–27 mm h^–1^ in response to limiting or excessive photon fluence rates (Hopkins, 1964; Round, 1971; Perkins et al., 2010).

Besides light, photosynthetic organisms are also dependent on temperature as photosynthesis, respiration and growth are all controlled by this physical factor (Raven and Geider, 1988; Tcherkez et al., 2006). In the shallow coastal area of the Baltic Sea, especially, water surface temperature naturally fluctuates due to seasonality and anthropogenic climate change leads to a warming of up to 0.6 K per decade exceeding the global ocean average by a factor of three (Reusch et al., 2018). Temperature strongly affects EPS production and excretion of temperate benthic diatoms (Wolfstein and Stal, 2002; Aslam et al., 2018), while growth is not influenced in a temperature range between 10 and 30°C (Scholz and Liebezeit, 2012). Net primary production of three benthic diatoms from the southern Baltic Sea increased with decreasing temperatures, and these data were explained by rising contents of photosynthetic enzyme molecules, such as ribulose-1.5-bisphospate carboxylase oxygenase (RuBisCO) (Mortain-Bertrand et al., 1988; Woelfel et al., 2014). Such a quantitative strategy guarantees to overcome any metabolic constraints imposed by low temperatures, and RuBisCO was identified as the key enzyme involved in such temperature acclimation, while β-carboxylases (e.g., PEP carboxykinase) played a minor role (Mortain-Bertrand et al., 1988). Therefore, the photosynthetic carbon assimilation is controlled by temperature-dependent enzymes (Falkowski and Raven, 1997). The influence of temperature on photosynthesis of benthic diatoms in the Baltic Sea is unstudied (except: Woelfel et al., 2014).

Shallow coastal areas are influenced by terrestrial runoff and may be affected by submarine ground water discharge (Jurasinski et al., 2018). The poor water exchange with the North Sea as well as the influx of agricultural fertilizers and other terrestrial residues has led to eutrophication of the Baltic Sea (Rönnberg and Bonsdorff, 2004). Although the Baltic Sea is characterized as a microtidal ecosystem, strong north-west winds can raise the water level and trigger storm floods at the southern coast. These atmospheric conditions can lead to flooding of coastal peatlands, which contain and store large amounts of carbon (Jurasinski et al., 2018). Peatlands in general store up to 30% of the global carbon by just covering 3% of the entire terrestrial area (Grützmacher, 2009). Coastal peatlands might be connected to the shallow water zone by underground peat layers and submarine groundwater discharge (Jurasinski et al., 2018), both facilitating the exchange of organic material, minerals and nutrients.

Dissolved organic compounds can be used by heterotrophic organisms to fuel their metabolism. Some microalgae are able to change their metabolic pathways from autotrophy to mixotrophy or even heterotrophy under constant darkness and use of organic compounds as a source of energy (Saks, 1983). Benthic diatom species such as *Nitzschia* spec. increase their production under mixotrophic conditions (Kitano et al., 1997). Heterotrophy in diatoms can either be attributed to a lack of photosynthetic pigments (obligate) or to a temporal separation of photosynthesis and respiration (facultative) like *Cyclotella cryptica* (Villanova et al., 2017). Many diatoms are using a heterotrophic pathway in order to maintain their photosynthetic ability under light-limited conditions, and hence the supply of organic carbon (e.g., glucose, acetate) is essential for long-term survival, for example, when buried in deeper sediment layers after a storm. However, the influence of organic substances from peatlands on benthic diatom growth has not yet been investigated in the southern Baltic Sea.

In the frame of the DFG Research Training Group ‘Baltic Transcoast’^1^ a representative coastal site was established as model for transport and transformation processes in the marine and the terrestrial part of the coast, and for evaluating the influence by water and matter inputs from the respective other coastal domain. The study area is the nature reserve “Heiligensee und Hütelmoor,” which is located as coastal fen next to the Baltic Sea near Rostock, Mecklenburg-Western Pomerania, northeastern Germany, on a transition zone between Atlantic maritime and continental climate (Jurasinski et al., 2018). Since the early 2000s the Hütelmoor is part of a restoration project attempting to reach the original condition as a coastal peatland. Therefore, coastal dunes and protection measures separating the Baltic Sea from the peatland will not be maintained. It is anticipated that the natural impact of the Baltic Sea including episodic flooding will re-establish in the near future, most probably resulting in repeated input of brackish waters (on decadal time scales) with possible consequences for hydro-physical and biogeochemical processes (Jurasinski et al., 2018). This will probably increase the exchange and material flows between both systems. In January 2017 a storm flood event already carried brackish water from the Baltic Sea into the Hütelmoor resulting in increasing water levels and salinity (Miegel et al., 2017). Just recently, on 2nd January 2019, another storm flood event caused a break of the main dune and Baltic Sea water flushed into the Hütelmoor. Measurements of salinity have proven saline water intrusion of over 1 km into the peatland (Jurasinski, personal communication), most probably with ecological consequences for the peatland and the exchange processes along the terrestrial-marine gradient. In addition, due to the disappearance of the protective sand dune even modest storm floods exceeding the thresholds of 0.5–1.00 m above sea level will lead to an increasing number of flooding events (Jurasinski et al., 2018). Additionally, there is an underground connection between the Hütelmoor and the shallow water zone as parts of the peat layer are stretching into the Baltic Sea (Jurasinski et al., 2018). Particularly dissolved organic compounds (DOM) of the peat layer may impact biological activities in the shallow coastal Baltic Sea area (Jurasinski et al., 2018), such as microphytobenthic primary production. DOM was found to be released in higher concentrations from terrestrial peat compared to the outcropping peat layers in the coastal Baltic Sea (Jurasinski et al., 2018). Since ecophysiological traits of clonal benthic diatoms from the Baltic Sea are almost unstudied, this study focused on photosynthesis and respiration of various diatom cultures isolated from the shallow coastal benthos of the southern Baltic Sea in proximity to the Hütelmoor. Furthermore, the impact of Hütelmoor water on growth of unialgal benthic diatoms under light and dark conditions was tested to identify if potential organic carbon sources may support heterotrophy.

## Materials and Methods

### Study Site and Cultures

Benthic diatom strains were isolated from undisturbed sediment cores sampled in nearshore sandy habitats of the Baltic Sea, north east of Rostock in close vicinity to the Hütelmoor (54°13.005′N, 12°9.051′E) at a water depth of 3–6.5 m. Water temperature ranges from 0–3°C during winter up to 20–23°C in summer, and salinity varies between 10 and 15 S_A_ (H. Lippert, unpublished data). This non-tidal system, however, is very dynamic concerning diatoms occurrences, due to strong mixing of the upper sediment layer at the coast line as well as vertical and horizontal exchange between the land and sea including wind, waves and lateral density gradients (Jurasinski et al., 2018).

From the sediment cores eight diatom species were isolated according the methodological approach of Stachura-Suchoples et al. (2016). The species included *Actinocyclus* octonarius Ehrenberg (1837), *Melosira moniliformis* (O. F. Müller) Agardh (1824), *Halamphora* sp. 1, *Halamphora* sp. 2, *Navicula perminuta* Grunow (1880), *Navicula phyllepta* Kützing (1844), *Nitzschia dubiiformis* Hustedt (1939) and *Nitzschia pusilla* Grunow (1862), and their morphological features, ecology and identification method (morphology, molecular genetics) are summarized in Table 1. Strains were morphologically identified using the taxonomic literature of Snoeijs and Vilbaste (1994), Snoeijs and Potapova (1995), Lange-Bertalot (2000, 2013), and Krammer and Lange-Bertalot (2007, 2010) and were compared to those in the database *Algae Base*^2^ for recent nomenclature. In addition, *N. phyllepta* was molecular-genetically identified according to the protocol of Zimmermann et al. (2011) using the highly variable V4 region of the SSU rRNA, while the *rbc*L gene was used for *A. octonarius*, *M. moniliformis*, *Halamphora* sp. 1, *Halamphora* sp. 2, *N. perminuta*, and *N. pusilla* following Abarca et al. (2014) to at least the genus level. *N. dubiiformis* could not be molecular-taxonomically identified. All sequences were submitted to the National Center for Biotechnology Information (NCBI) under following accession numbers: *rbc*L, *Actinocyclus octonarius*, MN097770, *Halamphora* sp. 1, MN097771, *Navicula perminuta* MN097772, *Nitzschia pusilla*, MN097773, *Melosira moniliformis*, MN097774, *Nitzschia dubiiformis*, MN097775; V4 SSU, *Halamphora* sp. 2, MN097879, *Navicula phyllepta* MH794232.

**TABLE 1:** Morphological and ecological characteristics of eight different diatom strains and identification method (IM) marked with G (genetical) or M (morphological); ^*^ marks only genus level.

**Isolate**	**Light microscopy image**	**SEM image**	**Size**	**Ecology**	**IM**
*Actinocyclus octonarius*, Ehrenberg (1837)	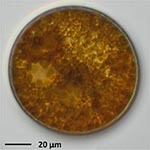	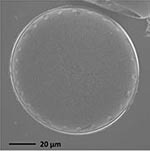	Diameter: 89.04 – 99.77 μm Areolae: 6 – 8 in 10 μm	Pelagic^2^, solitary^2^, cosmopolitan in marine plankton and coastal zone sediments^5^	M and G^*^
*Melosira moniliformis*, (O. F.Müller) C. Agardh, 1824	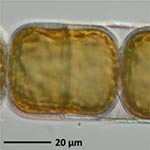	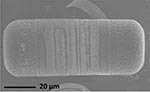	Diameter: 35.64 – 46.91 μm Striae: 10 – 12 in 10 μm	Widespread in brackish and marine coastal waters^5^	M andG
*Halamphora* sp. 1, Kützing, 1844	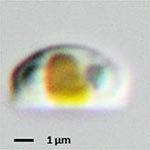	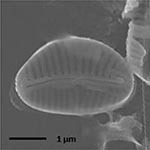	Length and width: < 5 μm	Cosmopolitan^3^	G^*^
*Halamphora* sp. 2, Kützing, 1844	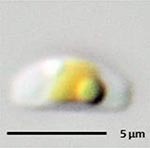	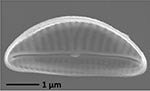	Length and width: < 5 μm	Cosmopolitan^3^	M
*Navicula perminuta*, Grunow in van Heurck, 1880	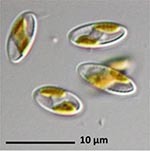	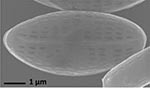	Length: 5.96 – 8.53 μm Width: 2.08 –3.56 μm Striae: 14 – 20 in 10 μm Areolae: 33 in 10 μm	Cosmopolitan^1,3^, locally abundant in brackish zones of rivers and along the coast^3,6^	M andG
*Navicula phyllepta*, Kützing, 1844	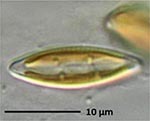	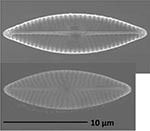	Length: 15.09 – 18.06 μm Width: 4.49 – 6.73 μm Striae: 14 – 20 in 10 μm Areolae: ca. 45 in 10 μm	Epipelic^2^, solitary^2^, motile^2^, brackish water of coastal regions^6^, cosmopolitan^3^	M and G
*Nitzschia dubiiformis*, Hustedt, 1939	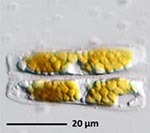	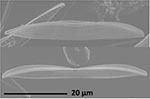	Length: 37.5 – 49.31 μm Width: 3.75 – 6.16 μm Striae: ca. 40 in 10 μm Fibulae: 12 –18 in 10 μm	Epipelic and epilithic^2^, solitary^2^, motile^2^, cosmopolitan at marine coasts^4^	M
*Nitzschia pusilla*, Grunow, 1862	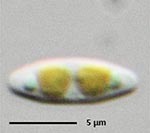	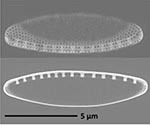	Length: 9.83 – 10.31 μm Width: 2.43 – 3.67 μm Striae: 43 – 55 in 10 μm Fibulae: 14 - 20 in 10 μm	Epipelic^2^, solitary^2^, motile^2^, cosmopolitan^6,4^	M and G

*Light microscopic images show the color of the living diatoms and scanning electron microscope (SEM) images show the typical shapes of the frustules. ^1^Snoeijs and Vilbaste (1994); ^2^Snoeijs and Potapova (1995); ^3^Krammer and Lange-Bertalot (2007); ^4^Krammer and Lange-Bertalot (2010); ^5^Lange-Bertalot (2000); ^6^Lange-Bertalot (2013).*

The diatom isolates were maintained as unialgal, but not axenic stock cultures in Guillard’s f/2 medium (Guillard and Ryther, 1962; Guillard, 1975) enriched with metasilicate (Na_2_SiO_3_ ∙ 5 H_2_O; 10 g 100 ml^–1^) at a final concentration of 0.6 mM. All media were set up with Baltic Sea water (∼12 S_A_) enriched with additional sea salt (hw Marinemix professional, hw Wiegandt Aquaristik, Krefeld, Germany) to achieve always a final salinity of 15 S_A_. Cultures were kept at 20°C at a photon fluence density of 30–50 μmol photons m^–2^ s^–1^ under a 16:8 h light:dark cycle (herein referred to as culture conditions). Using this cultivation approach typically resulted in low bacteria numbers ranging of maximum 0.05–0.2% of the diatom biomass.

### Light Response Curves (*PE*-Curves)

Light response (*PE*) curves were measured with a self-constructed *PE*-Box using 3 water-tempered DW1 oxygen electrode chambers each placed on a magnetic stirrer (Hansatech Instruments, King’s Lynn, United Kingdom) that were kept at a constant temperature of 20°C. Oxygen concentration in the chambers were measured with oxygen dipping probe DP sensors (PreSens Precision Sensing GmbH, Regensburg, Germany) connected to a fiber optic oxygen transmitter via optical fibers (Oxy 4-mini meter, PreSens Precision Sensing GmbH, Regensburg, Germany). Chambers were closed with an air-tight lid. Calibration and measurements were controlled and logged with the PreSens software OXY4v2_30 compatible to the optical transmitter. Before measurements, a two-point calibration (0 and 100% oxygen saturation) was carried out using culture medium.

Three replicates of 3 ml pre-incubated log phase suspension of each diatom culture were filled into each cuvette. To avoid carbon deficiency during measurements, sodium bicarbonate (NaHCO_3_, 2 mM final concentration) was added to each cuvette. Diatoms were exposed to ten increasing light levels ranging from 0 to 1249 μmol photons m^–2^ s^–1^ of photosynthetically active radiation (PAR) generated by LEDs (LUXEON Rebel1 LXML-PWN1-0100, neutral-white, Phillips, Amsterdam, Netherlands) implemented into the *PE*-Box. Light levels were measured with a spherical light sensor (light meter LI-250, LI-COR, Lincoln, United States) placed directly into the cuvette prior to each measurement. Measurements started with a respiration phase of 30 min in the dark followed by a 10 min photosynthesis phase for each light level. The first and last minute of each phase were not included in the calculations.

P-I curve data were calculated and fitted by the mathematical photosynthesis model of Walsby (1997). The different photosynthetic parameters were estimated from the least-square regression curves fitted to the measured values with the solver function of MS Office excel 2013. From these curves the maximum rate of net primary production (NPP_max_), respiration (R), light utilization coefficient (α), photoinhibition coefficient (β), light saturation point (I_k_) and the light compensation point (I_c_) were calculated.

After each *PE*-curve diatom suspension from each cuvette was filtered onto an individual Whatman GF/6 glass fiber filter (Ø 25 mm) for chlorophyll *a* measurements. Chlorophyll *a* was extracted using 3 ml of 96% ethanol, thoroughly vortexed and incubated for 24 h at 4°C in the dark. Extracts were centrifuged (Heraeus Megafuge 1.0R, Hanau, Germany) for 10 min at 1844 × *g* to decrease turbidity and the supernatant was measured at an extinction of 665 and 750 nm with a spectrophotometer (Shimadzu UV-2401 PC, Kyoto, Japan). Chlorophyll *a* was calculated according a protocol of the Baltic Marine Environment Protection Commission (1988; equation 1).

(1) $$\mu \,g\,C\,h\,l\,a\times l^{-1}=\frac{\left(E_{665}-E_{750}\right)\times v\times 10^{6}}{83\times V\times d}$$

given *v* as extraction volume (ml), *d* as cell length (cm) and *V* as volume of filtered suspension (ml).

### Temperature-Dependent Photosynthesis and Respiration

The effect of rising temperatures on respiratory oxygen consumption and photosynthetic oxygen evolution in the eight diatom isolates was followed between 5 and 40°C in 5°C increments using the oxygen optode system described above and the methodological approach of Karsten et al. (2010). Beginning with 5°C, all samples were incubated at each experimental temperature for 20 min in the dark before respiration was monitored for an additional 10 min followed by photosynthesis for another 10 min under saturating 267 ± 7 μmol photons m^–2^ s^–1^ PAR. This photon fluence rate was kept constant for all photosynthesis measurements. After measurement of the photosynthetic oxygen evolution, temperature was increased by 5°C, and after reaching the new temperature in the thermostatic chamber, a new incubation period was started. The oxygen consumption and production per time unit was referenced to the concentration of total Chl *a* per sample as described above. The final photosynthetic and respiratory rates were fitted using the widely applied model of Blanchard et al. (1996), which was originally developed to quantify temperature effects on photosynthesis in microphytobenthic communities. The resulting temperature-response curves were further quantitatively characterized by estimating various descriptive statistics such as minimum, maximum and optimum temperature of photosynthesis and respiration as well as the so-called “performance breath.” The “performance breath” is defined as arbitrary values for the 80 or 20% temperature range which equals “good” or “sufficient” photosynthesis/respiration, respectively, according the concept of Eggert et al. (2003). These authors introduced the “performance breath” to better distinguish between eurythermal and stenothermal algae.

In order to deeper characterize the photosynthetic and respiratory rates as function of increasing temperature the respective activation energies (*E_*a*_*) were calculated according the Boltzmann-Arrhenius function (Padfield et al., 2016).

### Diatom Growth in Different Media

The effect of Hütelmoor water on diatom growth (cell counts) was studied using the strains *A. octonarius* and *N. dubiiformis*. Both species were maintained in four different media (sterile filtered): (I) Baltic Sea water (S_A_ 15), (II) Baltic Sea water enriched with f/2 + Si (as described above; S_A_ 15), (III) Hütelmoor water salted-up with hw Marinemix professional sea salt (S_A_ 15), and (IV) salted Hütelmoor water (see III) additionally enriched with f/2 + Si (S_A_ 15). All media differed in their nutrient and organic component concentrations (Table 2). All experiments were conducted for 10 days under light (30 – 50 μmol photons m^–2^ s^–1^; 16:8 h light:dark) and in parallel under dark conditions. Prior to each experiment both diatom cultures were grown in fresh culture medium for 10 days to establish log phase biomass. Afterward, Erlenmeyer flasks were filled with the respective medium and inoculated with an always equal amount of cells per ml of the respective stock culture. Algal growth was determined in three replicate 3 ml samples preserved in Lugol’s iodine solution (Throndsen and Sournia, 1978) that were taken at the beginning as well as at the end of the experiment. Samples were counted in sedimentation chambers (area: 31.42 mm^2^) using an inverted microscope (Olympus IX70, Olympus, Hamburg, Germany) following the guidelines of Utermöhl (1958), high-density samples were counted up to 400 cells per sample. Cell number was always calculated per milliliter volume.

**TABLE 2:** Analysis of SAC_254_, salinity, phosphate (PO_4_^3–^), ammonium (NH_4_^+^), and nitrate (NO_3_^–^) in μmol/L for respective medium Baltic Sea water (Baltic), f/2 enriched Baltic Sea water (Baltic and f/2), salted Hütelmoor water (salted), and f/2 enriched Hütelmoor water (salted and f/2).

**Medium**	**PO_4_^3–^ μmol/L**	**NH_4_^+^ μmol/L**	**NO_3_^–^ μmol/L**	**N:P**	**SAC_254_ (1/m)**	**Salinity (S_A_)**
Baltic Sea	1.5	0.65	0.64	0.87	13	15.0
Baltic Sea + f/2	23.7	0.73	943	39.82	–	15.0
Hütelmoor	34.8	12.53	139	4.35	283	15.0
Hütelmoor + f/2	62.8	9.39	929	14.94	–	15.0

Each medium was filtered through a 0.45 μm cellulose acetate membrane filter (Sartorius, Goettingen, Germany) for analysis of phosphate (PO_4_^3–^), nitrate (NO_3_^–^) and ammonium (NH_4_^+^). PO_4_^3–^ and NO_3_^–^ were measured using a continuous-flow analyzer (Alliance Instruments, Salzburg, Austria) following the manufacturer’s protocol for both compounds. NH_4_^+^ was measured manually at 630 nm using a spectrophotometer after reaction of samples with a mixed phenol solution (3.5 g phenol and 0.04 g nitroprusside sodium dissolved in 100 ml demineralized water) and Trion solution (0.25 g Trion filled up to 100 ml citrate buffer solution) (DIN 38406 E5-1). The spectral absorption coefficient at 254 nm (SAC_254_) was determined as a proxy for dissolved organic compounds in the applied growth media using a spectrophotometer (Shimadzu UV-2401 PC, Kyoto, Japan). Furthermore, a visual aquarium quick assay for silicate was performed (JBL, Neuhofen, Germany) according the manufacturer’s protocol. The amount of silicate was categorized in nine color shades from <0.1 to >6.0 SiO_2_ mg l^–1^.

### Statistical Analysis

All calculations were undertaken using Microsoft Office Excel (2013). All values shown represent the mean value of three replicates. Significance levels were calculated using one-way ANOVA followed by a *post hoc* Tukey’s honest significant difference test (critical *p*-value < 0.05). Prior to the analysis, data were tested for normality using the Shapiro–Wilk’s test and for homogeneity of variances using Levene’s test. Statistical analysis of the data was performed using SPSS Statistics 22 (IBM, Armonk, NY, United States).

## Results

### *PE*-Curves

**FIGURE 1:**
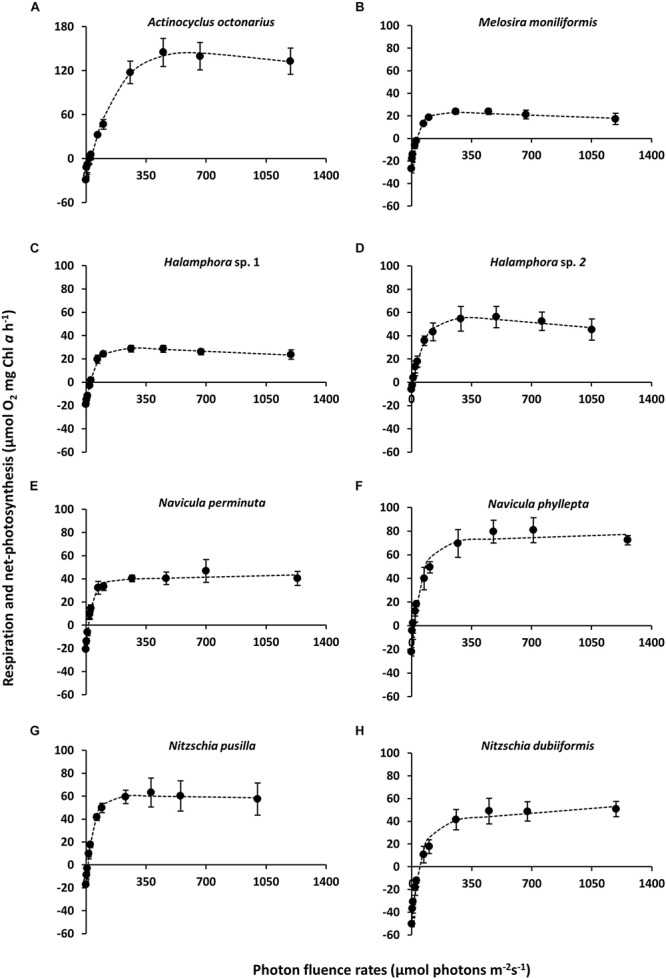
**(A–H)** Photosynthesis and respiration rates (μmol O_2_ mg^–1^ Chl *a* h^–1^) in relation to increasing photon flux density (μmol photons m^–2^s^–1^) of eight diatom cultures – kept at 20°C in f/2 Baltic Sea water medium, 15S_A_ – measured by oxygen evolution with optodes. Data represent mean values ± SD (*n* = 3). **(A)** *Actinocyclus octonarius*, notice differently sized axis; **(B)** *Melosira moniliformis*; **(C)** *Halamphora* sp. 1; **(D)** *Halamphora* sp. 2; **(E)** *Navicula perminuta*; **(F)** *Navicula phyllepta*; **(G)** *Nitzschia dubiiformis*; **(H)** *Nitzschia pusilla.*

The benthic diatoms studied showed species-specific respiratory oxygen consumption rates in the dark as well as photosynthetic oxygen evolution rates with increasing photon fluence rates up to ∼1250 μmol photons m^–2^ s^–1^ (Figures 1A–H). A potential contribution by diatom-associated bacteria to the oxygen signals can be neglected because of their always low cell numbers (less than 0.05–0.2% of diatom biomass) during exponential growth phase and during experimentation. Using the photosynthetic model of Walsby (1997), characteristic *PE*-curve parameters were calculated (Table 3). The dark respiration rates in most species were in a similar range (−17.1 to −29.2 μmol O_2_ mg^–1^ Chl *a* h^–1^), except in *Halamphora* sp. 2 and *N. dubiiformis* that exhibited much lower or higher rates with −6.1 and −50.2 μmol O_2_ mg^–1^ Chl *a* h^–1^, respectively (Table 3). A similar broad range was measured for the maximum photosynthetic rates (NPP_max_ 23.1 to 144.1 μmol O_2_ mg^–1^ Chl *a* h^–1^) in the 8 diatom species, while the light utilization coefficient (α), which reflects photosynthetic efficiency, was with 0.88 to 1.88 μmol O_2_ mg^–1^ Chl *a* h^–1^ (μmol photons m^–2^ s^–1^)^–1^ rather similar (Table 3). Light compensation points (I_c_) were between 7.3 and 45.5 μmol photons m^–2^ s^–1^, and the light saturation points (I_k_) ranged from 31.6 to 69.8 μmol photons m^–2^ s^–1^ in 7 out of 8 species (Table 3). In contrast, *A. octonarius* exhibited with 151.3 μmol photons m^–2^ s^–1^ a 2–5 times higher I_k_ value. The Walsby model fit indicated that 5 out of 8 species showed slight photoinhibition under the highest photon fluence rate tested [β: −0.01 to −0.03 μmol O_2_ mg^–1^ Chl *a* h^–1^ (μmol photons m^–2^ s^–1^)^–1^], while the remaining 3 species (*N. perminuta*, *N. phyllepta*, *N. dubiiformis*) were not photoinhibited at all (Table 3). NPP_max_ to respiration ratios ranged from 0.8 to 9.1 (Table 3).

**TABLE 3:** Parameter of respective *PE*-curves (Figures 1A–H) of eight diatom cultures (*n* = 3) kept at 20°C in a f/2 Baltic Sea water medium, 15S_A_.

**Isolates**	**NPP_max_ (μmol O_2_ mg^–1^ Chl *a* h^–1^)**	**Respiration (μmol O_2_ mg^–1^ Chl *a* h^–1^)**	**α (μmol O_2_ mg^–1^ Chl *a* h^–1^) (μmol photons m^–2^ s^–1^)^–1^**	**β (μmol O_2_ mg^–1^ Chl *a* h^–1^) (μmol photons m^–2^ s^–1^)^–1^**	**I_k_ (μmol photons m^–2^ s^–1^)**	**I_c_ (μmol photons m^–2^ s^–1^)**	**NPP_max_: Respiration**	
*Actinocyclus octonarius*	144.07±19.47⁢a	-29.21±1.30	1.15±0.14	-0.03±0.01	151.34±9.51	28.5±2.20	4.93±0.54
*Melosira moniliformis*	23.12±1.62⁢b	-26.96±3.55	1.26±0.18	-0.01±0.00	39.78±2.31	30.57±3.12	0.86±0.08
*Halamphora* sp. 1	29.31±2.68⁢bcd	-19.01±2.59	1.01±0.24	-0.01±0.00	48.05±5.52	24±2.29	1.54±0.07
*Halamphora* sp. 2	55.46±10.62⁢cde	-6.07±6.18	0.88±0.15	-0.01±0.01	69.75±7.96	7.32±3.35	9.14±0.22
*Navicula perminuta*	38.73±2.86⁢bcd	-20.84±4.81	1.88±0.58	0.01±0.01	31.61±2.22	13.49±1.26	1.86±0.08
*Navicula phyllepta*	70.81±7.89⁢e	-22.15±14.67	1.64±0.76	0.01±0.00	56.66±12.62	15.36±3.61	3.20±0.56
*Nitzschia dubiiformis*	38.60±12.08⁢b	-50.18±3.33	1.6±0.63	0.01±0.01	55.55±12.76	45.50±6.24	0.77±0.19
*Nitzschia pusilla*	60.12±12.03⁢d	-17.11±2.85	1.84±0.15	-0.02±0.00	41.93±9.32	10.50±0.45	3.51±0.19

*NPP_*max*_ represents the maximal oxygen production rate, alpha the initial slope of production in the light limited range, beta the terminal slope of production in extensive light range (photoinhibition), I_*k*_ the light saturation point, I_*c*_ the light compensation point. Data represent a mean value ± SD (*n* = 3). Different lowercase letters represent significance levels among all means as calculated by a one-way ANOVA (Tukey’s test, *p* < 0.05). NPP_*max*_: *F* = 29.576, df = 4, *p* < 0.001.*

### Temperature-Dependent Photosynthesis and Respiration

**FIGURE 2:**
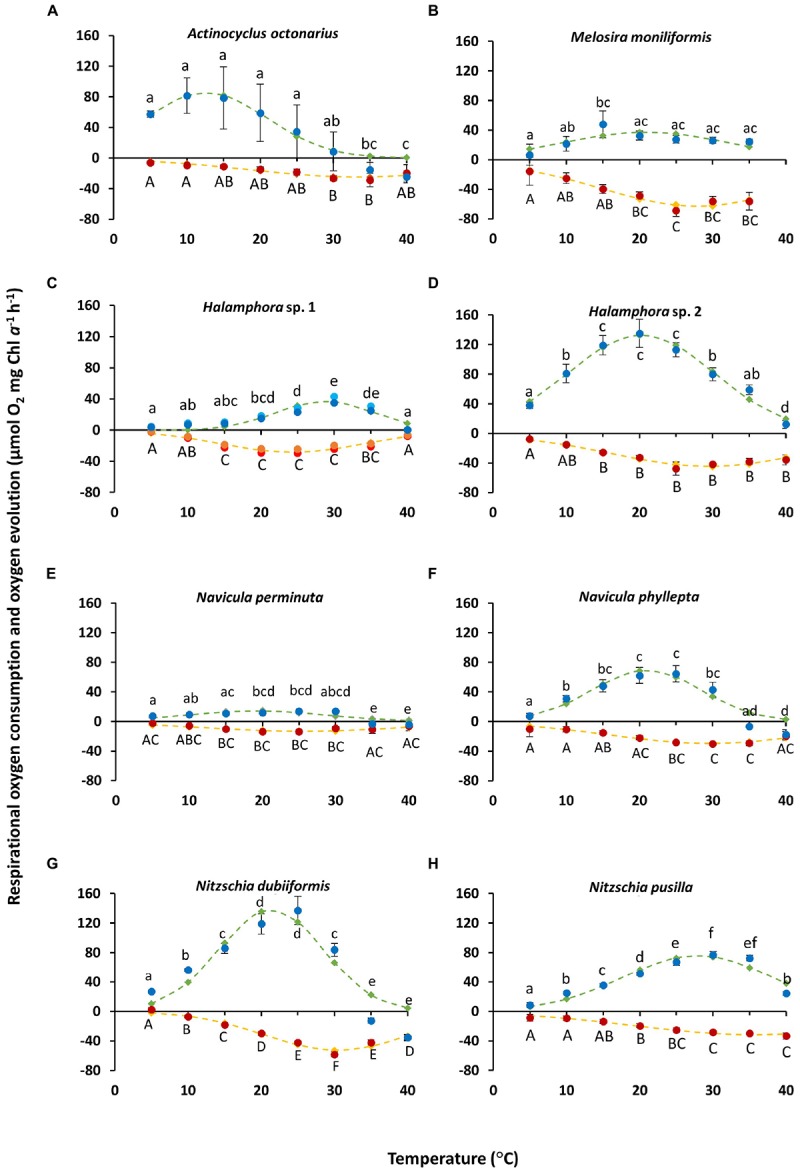
**(A–H)** Photosynthetic (blue) oxygen evolution at 266 ± 6.7 μmol photons m^–2^s^–1^ and respiratory (red) oxygen consumption in darkness of eight different diatom cultures as a function of increasing temperature. The measured data were fitted by the model of Blanchard et al. (1996) (photosynthesis: green dashed line; respiration: yellow dashed line). All cultures were kept in f/2 Baltic Sea water medium, 15_SA_. Data represent mean values ± SD (*n* = 3). Different lowercase (photosynthesis) and capital letters (respiration) indicate significantly means (*p* < 0.05; one-way ANOVA with *post hoc* Tukey’s test). **(A)** *Actinocyclus octonarius*; **(B)** *Melosira moniliformis*; **(C)** *Halamphora* sp. 1, notice values consist of *n* = 2 (represented each by different colors), **(D)** *Halamphora* sp. 2; **(E)** *Navicula perminuta*; **(F)** *Navicula phyllepta*; **(G)** *Nitzschia dubiiformis*; **(H)** *Nitzschia pusilla.*

The effect of increasing temperature from 5 to 40°C on net photosynthetic oxygen evolution and respiratory oxygen consumption revealed strong differences between both physiological processes and among the benthic diatoms studied (Figures 2A–H). Rising temperature stimulated photosynthesis and respiration up to a species-specific maximum followed by a decline under the highest temperature conditions. While optimum photosynthesis was measured between 10 and 30°C among all species studied, highest respiration occurred between 25 and 40°C (Figures 2A–H).

Photosynthetic oxygen evolution of *N. phyllepta* (Figure 2F) and *N. dubiiformis* (Figure 2G) significantly increased from 5 to 20°C/25°C, the latter temperatures reflecting the species-specific optimum. Further increase in temperature to 30°C was accompanied by a 30–40% decline in photosynthesis, while incubation at 35 and 40°C even led to complete inhibition as reflected in negative values for oxygen evolution. Respiratory oxygen consumption in both species increased between 5 and 30°C (Figures 2F,G). Further rise in temperature (35 and 40°C) led to a significant decrease in respiratory activity. The maximum photosynthetic oxygen evolution of *N. dubiiformis* was (137 μmol O_2_ mg^–1^ Chl *a* h^–1^) about twice of that in *N. phyllepta* (65 μmol O_2_ mg^–1^ Chl *a* h^–1^). In addition, the maximum respiratory oxygen consumption of *N. dubiiformis* (−58 μmol O_2_ mg^–1^ Chl *a* h^–1^) was also twofold higher compared to *N. phyllepta* (−30 μmol O_2_ mg^–1^ Chl *a* h^–1^) (Figures 2F,G).

The photosynthetic oxygen evolution in *Halamphora* sp. 2 (Figure 2D) and *N. pusilla* (Figure 2H) also increased with rising temperatures. While *Halamphora* sp. 2 showed a broad optimum between 15 and 25°C, the second species exhibited maximum photosynthesis at 30°C. Higher temperatures of 35 and 40°C led in both strains to a decline of photosynthesis, but not to complete inhibition (Figures 2D,H). Maximum photosynthesis of *Halamphora* sp. 2 was almost 1.7-fold higher (129 μmol O_2_ mg^–1^ Chl *a* h^–1^) compared to *N. pusilla* (77 μmol O_2_ mg^–1^ Chl *a* h^–1^). Respiratory oxygen consumption in both taxa displayed similar trends as respiration rates increased with rising temperatures up to −40 μmol O_2_ mg^–1^ Chl *a* h^–1^ at 25 and 30°C, respectively, and remained on this level with further rising temperatures up to 40°C (Figures 2D,H).

In contrast to the previously described species, *Halamphora* sp. 1 and *N. perminuta* (Figures 2C,E) exhibited generally much lower values of maximum photosynthetic oxygen evolution (14–39 μmol O_2_ mg^–1^ Chl *a* h^–1^). Both strains showed increasing photosynthesis rates with rising temperatures and optimum rates at 30°C, followed by a strong decline at 35°C (Figures 2C,E). At 40°C photosynthesis was completely inhibited. Respiratory oxygen consumption rose with increasing temperatures up to 30°C, followed by a slight decrease with further rising temperature (Figures 2C,E).

Photosynthetic oxygen evolution in *M. moniliformis* increased between 5 and 15°C up to 48 μmol O_2_ mg^–1^ Chl *a* h^–1^, with the optimum at 15°C (Figure 2B). Further rise in temperature was accompanied by a 45% decline in photosynthesis, which, however, remained constant between 20 to 40°C at 24–27 μmol O_2_ mg^–1^ Chl *a* h^–1^ (Figure 2B). Respiratory oxygen consumption linearly increased from 5 to 25°C up to −60 μmol O_2_ mg^–1^ Chl *a* h^–1^, followed by a slight decrease at 30 and 35°C (Figure 2B). Due to technical malfunctions, no measurements were made at 40°C for *M. moniliformis*.

*Actinocyclus octonarius* showed highest photosynthetic oxygen evolution at lower temperatures (5 to 25°C), followed by a strong to complete inhibition of photosynthesis between 30 and 40°C (Figure 2A). The respiratory oxygen consumption slowly increased from 5 to 30°C, with an optimum at 30–35°C, followed by a slight decline at 40°C (Figure 2A).

In addition, the optimum temperatures for photosynthesis and respiration were also calculated using the widely used model of Blanchard et al. (1996) (Figures 2A–H, green and yellow dashed lines), which provided more precise values. For five species (*M. moniliformis*, *Halamphora* sp. 2, *N. perminuta*, *N. phyllepta*, *N. dubiiformis*) optimum temperature for photosynthesis was calculated between 19.2 and 21.4°C which reflects the upper temperature range of their natural habitat (Table 4). While for *N. pusilla* and *Halamphora* sp. 1 higher temperature optima for photosynthesis between 27.9 and at 28.8°C were calculated, *A. octonarius* exhibited lower temperatures in the model (12.6°C) (Table 4). At the upper 80% percentile, modeled data of the optimum temperature for photosynthesis confirmed those of the measurements. In contrast, at the upper 20% percentile a broader temperature range was calculated by the model compared to the measurements. Calculation of the optimum temperature for respiration resulted in evenly distributed rates among the eight diatom strains, ranging from 24.3°C in *Halamphora* sp. 1 to 35.7°C in *N. pusilla* (Table 4). The model data confirmed the higher temperature requirement for respiration compared to photosynthesis, as well as a broader temperature tolerance as reflected in the upper 80% percentile and the upper 20% percentile (Table 4).

**TABLE 4:** Calculation of temperature effects on photosynthetic oxygen evolution **(A)** and respirational oxygen consumption **(B)** of eight tested benthic Baltic Sea diatom strains using the Blanchard et al. (1996) fit; additionally, the respective activation energy (*E_*a*_*) is given.

	**dO_2_ (μmol O_2_ mg^–1^ Chl *a* h^–1^)**			**Temperature (°C)**			**E_a_ (eV)**
	100%	>80%	>20%	100%	>80%	>20%	
**(A) Photosynthesis**							
*Actinocyclus octonarius*	85	>68.06	>17.01	12.6	7 – 18.2	(−2.5) – 27.6	0.47
*Melosira moniliformis*	37.17	>29.74	>7.43	20.9	13.2 – 28.6	(0.2) – (41.5)	1.37
*Halamphora* sp. 1	36.92	>29.54	>7.38	28.8	24.3 – 33.2	16.8 – (40.7)	0.62
*Halamphora* sp. 2	132.85	>106	>26.57	20.3	13.5 – 27	(2.0) – 38.4	0.59
*Navicula perminuta*	14.32	>11.46	>2.86	19.2	12.9 – 25.6	(2.2) – 36.2	0.22
*Navicula phyllepta*	68.97	>55.18	>13.79	21.0	15.9 -26.0	7.4 – 34.5	0.72
*Nitzschia dubiiformis*	138.11	>110.49	>27.62	21.4	16.6 – 26.1	8.5 – 34.2	0.58
*Nitzschia pusilla*	75.67	>60.54	>15.13	27.9	21.0 – 34.8	9.4 – (46.3)	0.60
**(B) Respiration**							
*Actinocyclus octonarius*	–25.07	<-20.06	<-5.01	33.4	23.5 – (43.4)	6.6 – (60.1)	0.38
*Melosira moniliformis*	–62.85	<-50.28	<-12.57	27.9	19.0 – 36.8	(3.9) – (51.8)	0.52
*Halamphora* sp. 1	–28.04	<-22.43	<-5.61	24.3	17.7 – 31.0	6.4 – (42.1)	0.98
*Halamphora* sp. 2	–44.64	<-35.71	<-8.93	29.5	20.5 – 38.5	5.3 – (53.6)	0.63
*Navicula perminuta*	–13.11	<-10.49	<-2.62	25.5	16.0 – 35.0	(−0.1) – (50.9)	0.67
*Navicula phyllepta*	–29.41	<-23.53	<-5.88	29.6	20.4 – 38.8	(4.8) – (54.2)	0.38
*Nitzschia dubiiformis*	–52.59	<-42.07	<-10.52	30.4	23.8 – 37.0	12.06 – (48.2)	1.92
*Nitzschia pusilla*	–31.35	<-25.08	<-6.27	35.7	24.6 – (46.8)	5.8 – (65.4)	0.39

*Data represent mean values (*n* = 3) of the maximum photosynthesis and respiration (100%) as well as the upper 80 and 20% percentile. Data in brackets represent temperature calculation exceeding measured temperature.*

**FIGURE 3:**
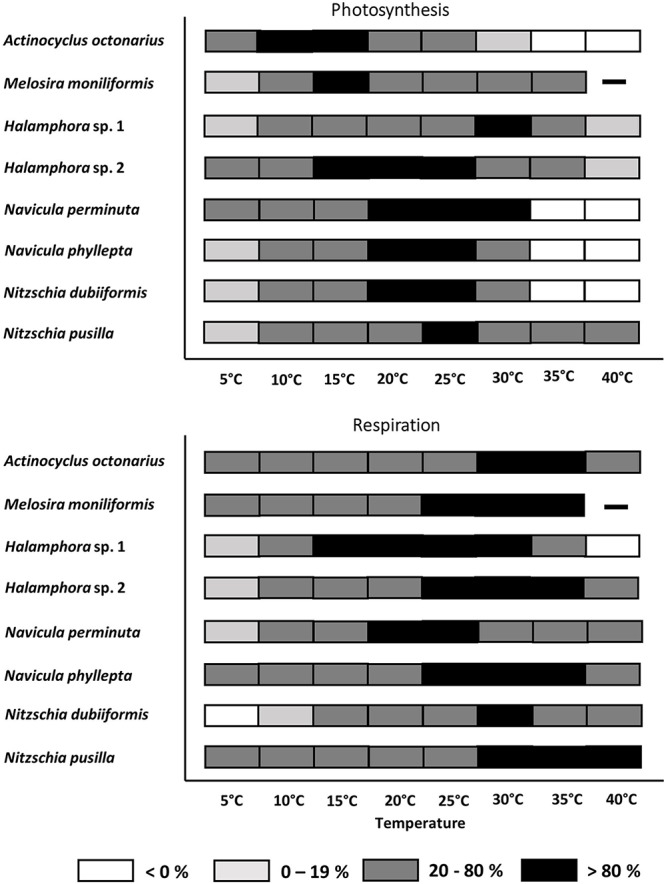
Effect of temperature on photosynthetic oxygen evolution and respirational oxygen consumption of eight tested diatom strains. Black symbols represents range of highest photosynthesis at > 80% percentile, dark gray symbols within 20 and 80% percentile light gray symbols < 20% percentile and white symbols no photosynthesis or respiration signal. Due to technical problems *Melosira moniliformis* could not be measured at 40°C. Data represent mean values (*n* = 3).

From the photosynthesis and respiration data in Figures 2A–H the species-specific temperature tolerance width for both physiological processes was calculated as percentiles of < 0%, 0–19%, 20–80%, and >80% of the optimum values (Figures 3A,B). All benthic diatoms showed a wide range of temperature tolerance for photosynthesis and respiration with almost all values surpassing the 20% percentile, and hence the eight species can be characterized as eurythermal organisms.

All benthic diatom species photosynthesized at 5°C, but only 3 out of 8 taxa were also capable to do so at 40°C (both *Halamphora* strains, *N. pusilla*). Half of the studied species exhibited photosynthesis up to 30°C, while at 35°C and 40°C complete inhibition was determined (Figure 3A). *Melosira moniliformis* showed photosynthesis up to 35°C. *Actinocyclus octonarius* and *M. moniliformis* had their photosynthetic optima at lower temperatures between 10 and 15°C, while all other species between 15 and 30°C. Both *Halamphora* species markedly differed in their photosynthesis-temperature patterns. While *Halamphora* sp. 1 showed a distinct optimum at 30°C, that of *Halamphora* sp. 2 shifted toward lower temperatures and was rather broad between 15 and 25°C (Figure 3A).

Except *N. dubiiformis* all other benthic diatom species respired at 5°C, and 6 out of 8 taxa were also capable to do so at 40°C. *Melosira moniliformis* and *Halamphora* sp. 1 showed respiration only up to 35°C (Figure 3B). Compared to photosynthesis, the upper percentile of >80% for respiration was always at higher temperatures, ranging from 15 to 40°C. The respiratory activity optimum of *Halamphora* sp. 2, *N. pusilla*, *N. phyllepta*, and *M. moniliformis* was between 25 and 35°C, partly overlapping with their photosynthesis optima. *Halamphora* sp. 1 revealed widest optimum respiration activity (>80% at 15–30°C) among the tested isolates. Overall, also concerning respiration all benthic diatom species can be characterized as eurythermal organisms. Thermal response calculations indicated species-specific activation energy (*E_*a*_*) for photosynthesis as well as for respiration among the tested benthic diatom species. *Navicula perminuta* showed lowest *E_*a*_* for photosynthesis (0.22 eV) and *M. moniliformis* exhibited the highest value (1.37 eV). Both *Halamphora* and both *Nitzschia* species had almost identical *E_*a*_* values ranging from 0.58 to 0.62 eV (Table 4). Activation energies for respiration were between 0.38 and 1.92 eV among all benthic diatom taxa. In contrast to photosynthesis both *Halamphora* and both *Nitzschia* species showed stronger differences in *E_*a*_* values for respiration (*Halamphora*: 0.63 and 0.98 eV; *Nitzschia*: 0.39 and 1.92 eV) (Table 4).

### Diatom Growth in Different Media

**FIGURE 4:**
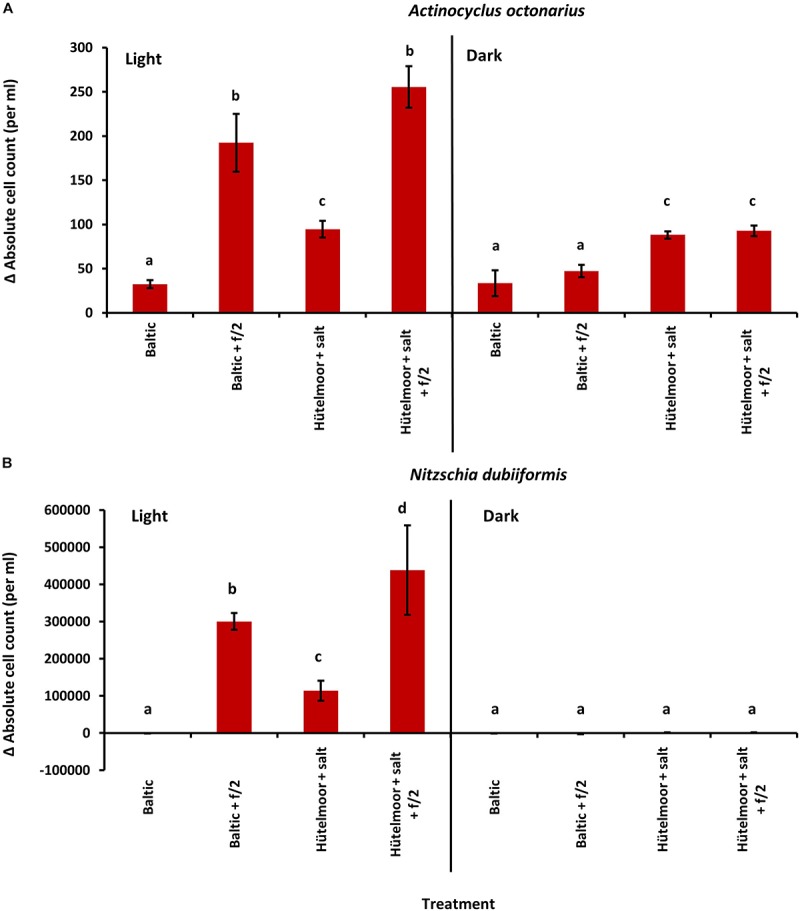
Absolute cell count change after 10 days cultivation from initial values of **(A)** *Actinocyclus octonarius* (initially approximately 40 cells per ml) and **(B)** *Nitzschia dubiiformis* (initially approximately 1400–4000 cells per ml) at 20°C under different light [light (16:8 h light dark cycle) and dark] and medium conditions. Data represent mean values ± SD (*n* = 3). Different lowercase letters indicate significantly different means (*p* < 0.05; one-way ANOVA with *post hoc* Tukey’s test).

In the growth experiments with *A. octonarius* the initial cell density for each treatment was with ca. 40 cells ml^–1^ highly similar, and after 10 days incubation an increase of cell number was observed in all media (Figure 4A). Cells grew under light and dark conditions, depending on the media used. Light treatment and incubation in media enriched with f/2 supplement resulted in highest increase in cells densities (Baltic Sea water 200 cells ml^–1^, Hütelmoor water 250 cells ml^–1^). Baltic Sea water without f/2 led to 67 cells ml^–1^ and Hütelmoor water without f/2 to 100 cells ml^–1^ under both light and dark conditions. In the dark, the addition of f/2 did not significantly increase cell density in the Baltic Sea water nor in the Hütelmoor medium compared to the pure medium (Figure 4A). Nevertheless, both Hütelmoor water media resulted in a two-fold increase of cell number in darkness compared to pure Baltic Sea water in the dark and in the light.

The initial cell numbers of *N. dubiiformis* differed significantly among treatments with 1.400 to 4.000 cells ml^–1^ (One-way ANOVA with *post hoc* Tukey’s test, *p* < 0.05). Cells grew only in the light, with significant differences depending on the media used (One-way ANOVA with *post hoc* Tukey’s test, *p* < 0.05) (Figure 4B). This species did not grow in pure Baltic Sea water. The highest increase in cell number was observed in the Hütelmoor water enriched with f/2 (400.000 cells ml^–1^), while Baltic Sea water enriched with f/2 resulted in about 300.000 cells ml^–1^. Hütelmoor water without f/2 stimulated cell growth up to 100.000 cells ml^–1^ (Figure 4B).

## Discussion

The eight benthic diatom species from the shallow coastal water zone of the southern Baltic Sea showed a broad photosynthetic performance at different photon fluence rates from 0 to 1250 μmol photons m^–2^ s^–1^ as well as at different temperatures from 5 to 40°C. These data point to rather high photo-physiological plasticity since in five out of eight species only minor photoinhibition was observed, while the remaining strains were not photoinhibited at all. In addition, the temperature tolerance was generally broad characterizing the benthic diatoms studied as eurythermal organisms. Nevertheless, strong species-specific ecophysiological response patterns could be identified, as well as a stimulating effect of Hütelmoor water on algal growth.

### Light and Temperature Effects on Photosynthesis and Respiration

The *PE*-curves of the investigated species revealed maximum net primary production over a wide range of photon fluence rates. Similar responses were reported by Colijn and Buurt (1975) on mixed benthic diatom populations from the Dutch Wadden Sea, which were dominated by few *Navicula* species exhibiting saturated photosynthesis between 135 and 810 μmol photons m^–2^ s^–1^ at 6 and 12°C without photoinhibition. Microphytobenthic assemblages from the intertidal zone of the west coast of Portugal, including *inter alia Nitzschia* and *Navicula* species, were also not photoinhibited when kept under increasing photon fluence rates up to 1700 μmol photons m^–2^ s^–1^ (Serodio et al., 2006). Although the important function of benthic diatoms in primary production in the shallow non-tidal waters of the Baltic Sea has been documented on a community level under different environmental settings (Wulff et al., 1997; Meyercordt and Meyer-Reil, 1999; Meyercordt et al., 1999), few studies exist on the underlying benthic diatom ecophysiology and ecological tolerances in these brackish habitats (e.g., Woelfel et al., 2014).

Woelfel et al. (2014) carried out similar experiments, measuring the photosynthetic responses of three benthic diatoms including *M. moniliformis* and *N. perminuta*, which, however, were isolated from a protected shallow-water area (15–30 cm water depth, 15–20 S_A_) approximately 65 km west of Hütelmoor. According to this study, maximum photosynthesis in both taxa was reached at 150 μmol photons m^–2^ s^–1^ and no photoinhibition up 600 μmol photons m^–2^ s^–1^ was found. However, NPP_max_ and the light-saturated range at 19°C and 24°C were two times higher compared to the respective taxa of the present study. Respiration of *N. perminuta* was similar to that in Woelfel et al. (2014), while it differed in *M. moniliformis* measurements. Respirational differences might be intuitively explained by a different degree of heterotrophic bacterial activity as cultures were not axenic. Any excretion of organic compounds by diatoms, which is known to be stimulated by high photon fluence rates, might potentially increase bacterial respiration, which would methodically lead to overall higher oxygen consumption signals (Epping and Jørgensen, 1996; Bohórquez et al., 2018). However, in the present study all diatom cultures showed always very low numbers of associated bacteria ranging from 0.05 to 0.2% of the diatom biomass, and hence we conclude that the bacterial influence on the oxygen signals can be neglected. Wolfstein and Stal (2002) found in a similar culture approach that bacterial biomass accounted for only 0.04% of the algal carbon and hence did not influence photosynthetic ^14^C-fixation experiments. In addition, the exudation of organic compounds by benthic diatoms such as *Cylindrotheca closterium* typically occurs after longer exposure to high photon fluence rates above 500–1000 μmol photons m^–2^ s^–1^, as a result of overflow metabolism due to limited internal storage capacity (Wolfstein and Stal, 2002). In contrast, all our photosynthesis experiments were rather short and during temperature treatment the maximum photon fluence rate did not exceed 270 μmol photons m^–2^ s^–1^, and hence C exudation seems unlikely.

An alternative explanation for the strong differences in ecophysiological response patterns among isolates of the same species might be related to ecotypic differentiation, which is considered as a genetically distinct geographic variety or population within a species that is adapted to specific environmental conditions. Ecotypic differentiation has been described for diatoms (e.g., Bailleul et al., 2010), but the designation of populations of a species as ecotypes (*sensu* Turesson, 1922) remains difficult. Differential physiological responses and a high degree of genetic heterogeneity among seasonally separated isolates of the pelagic diatom *Skeletonema marinoi* was documented and interpreted as genetic (ecotypic) differentiation of populations succeeding each other (Saravanan and Godhe, 2010). High alpha values in combination with low light compensation points between 7 and 45 μmol photons m^–2^ s^–1^ for all eight isolates indicate high photosynthetic efficiency under low light conditions. Different abiotic factors, such as clouds and turbidity, decrease light availability in the shallow water habitat of the coastal Baltic Sea. Decreasing photon fluence rates typically induce an increase in the amount of photosynthetic pigments such as Chl *a*, Chl *c* and fucoxanthin (Falkowski and Owens, 1980), which enhance photosynthesis under low irradiances. Although, in numerous macroalgae low-light adaptation of photosynthesis is usually coupled to strong photoinhibition under higher photon fluence densities (Hanelt et al., 1997; Bischof et al., 1998; and references therein), no indication of such expected photoinhibition could be noted in the eight benthic diatoms investigated, which may be interpreted as high photo-physiological plasticity. This high physiological plasticity can be linked to the diatom evolution. Photosynthesis in diatoms occurs in chloroplasts, which are endosymbiotic organelles derived from cyanobacteria. Since these eukaryotes acquired photosynthesis via endosymbiosis of another eukaryotic alga that already had plastids, the resulting organisms are chimeras with major genomic contributions from two or even more sources (Delwiche, 2007). As a consequence of this genomic mixing the diatom lineage with specific and often unique physiological and biochemical properties evolved. Different species of diatoms are characterized by a complex combination of genes and metabolic pathways acquired from a variety of sources such as red algae, green algae, a chlamydial parasite and bacteria (Armbrust, 2009). The consequences of this genetic mixture are reflected in specific biochemical capabilities, which might be even species-specific.

Diatoms are well known to adjust quickly to fluctuating light regimes, such as those found in the Baltic Sea (Wagner et al., 2006; Lavaud et al., 2007). However, excessive light can lead to photodamage due to the formation of reactive oxygen species (ROS) (Apel and Hirt, 2004; Choudhury et al., 2017), causing damage of biomolecules involved in the photosynthetic machinery.

To minimize such damage on a short-term scale, benthic diatoms use two main mechanisms: (1) vertical movement in the sediment (restricted to pennate diatoms) (Perkins et al., 2010) and (2) dissipation of excessively absorbed energy through photoprotection (Nymark et al., 2009 and references therein). Using the first strategy, diatoms can escape excessive light conditions by moving downward into the sediment (Cohn and Disparti, 1994; Mouget et al., 2008; Perkins et al., 2010). This motility allows the pennate raphid species from this study (*Halamphora* sp. 1, *Halamphora* sp. 2, *N. perminuta*, *N. phyllepta*, *N. dubiiformis*, and *N. pusilla*) to avoid high light exposure. Vertical movement in nature is efficient in terms of energy requirements for protection (Perkins et al., 2010). In the present study, however, vertical movement was prevented by constant stirring of the cultures during experimentation, and hence this protective mechanism cannot explain the lack of photodamage. As no or only very little photoinhibition was detected in each diatom species at even high photon fluence rates of up to 1250 μmol photons m^–2^ s^–1^, it is likely that these protists used rather the second mechanism involving molecular structural change. One of the most important protection mechanisms in diatoms is the dissipation of excessive excitation energy as heat in the light-harvesting complexes of the photosystems (Goss and Lepetit, 2015). This process requires a structural change of the antenna complexes that are typically optimized with regard to efficient light-harvesting. In addition, the conversion of xanthophyll diadinoxanthin to diatoxanthin during irradiation and the reverse reaction in darkness is involved in photoprotection in diatoms (Lavaud et al., 2007; Bojko et al., 2019). The combination of genotypic differentiation and adaptive mechanisms of benthic diatoms might explain the high photo-physiological tolerance necessary to cope with strongly fluctuating light conditions in the shallow coastal water zone (Falkowski and Owens, 1980; Barrett and Schluter, 2008). In the shallow water zone of the Baltic Sea, the underwater light conditions are highly dynamic and are regularly changing because of meteorology, waves and currents, neglecting differently distributed diatom species in the depth of 3–6.5 m in this area. The 1% depth for incident PAR has been estimated at 6.24 m at a comparable station (Zingst, 45 km further east) (Sagert and Schubert, 1999), i.e., that during summer c. 20 μmol photons m^–2^ s^–1^ reach the sea floor at 6–7 m depth.

Besides light also temperature controls photosynthesis in diatoms via numerous photosynthetic enzymes, such as Ribulose-1,5-bisphosphat carboxylase-oxygenase (RuBisCO) (Tcherkez et al., 2006). At higher temperatures, the affinity of RuBisCO to CO_2_ decreases compared with the affinity to oxygen, resulting in a lower carboxylase and a higher oxygenase activity, respectively (Young et al., 2014). The seasonal water temperature in the southern coastal Baltic Sea varies from 1.3°C in winter up to 21.4°C in summer (data for 2016, H. Lippert, unpublished). Experimental temperatures covered most of the natural conditions of the Baltic Sea as well as higher extremes to identify the upper thermal tolerance of the benthic diatoms. For seven of the isolates the optimal temperature for photosynthesis was within the range of their natural habitat (Figure 3), and most species could even well cope with temperatures up to 30°C. Although heat waves in the Baltic Sea are predicted to increase (Reusch et al., 2018), the investigated benthic diatoms will probably face no physiological problems as eurythermal organisms. Temperatures > 30°C, however, led to strong or even complete inhibition of photosynthesis. Increasing temperatures are directly linked to faster metabolic rates, resulting finally in an acceleration of RuBisCO deactivation (Crafts-Brandner and Salvucci, 2000). In addition, RuBisCO is adapted to the organism’s thermal environment. With rising temperature, this key enzyme is losing specificity for CO_2_ and O_2_, thereby getting less efficient (Tcherkez et al., 2006). Mortain-Bertrand et al. (1988) reported a seven-fold increase of RuBisCO activity of the diatom *Skeletonema costatum* at 3°C compared to 18°C, when grown for 3 months under these temperatures. Heat stress within the photosynthetic apparatus can cause instability of the photosystem II (PSII) during photosynthetic activity (Allakhverdiev et al., 2008). Even though moderate heat stress does not seriously damage the PSII, it can cause inhibition of repair mechanisms protecting the PSII (Allakhverdiev et al., 2008). Therefore, under heat stress the balance between damage and recovery processes as basis for acclimation gets disturbed (Adir et al., 2003; Mohanty et al., 2007; Murata et al., 2007), but also all other biochemical processes involved in photosynthesis. The effect of increasing temperatures on photosynthetic oxygen production and respiratory oxygen consumption in the eight benthic diatom strains showed strong differences in the temperature requirements of both physiological processes. This is not unusual since various authors clearly documented differential temperature-dependence of respiration and photosynthesis for different terrestrial and marine organisms (Allen et al., 2005; López-Urrutia et al., 2006). While Allen et al. (2005) reported a 16-fold increase in respiration over the temperature range 0–30°C due to the temperature dependence of ATP synthesis in respiratory complexes, only a fourfold increase in photosynthesis over the same temperature gradient occurred because of the temperature dependence of RuBisCO. While most diatom isolates exhibited optimum photosynthesis between 10 and 30°C, respiration was highest between 20 and 40°C. In addition, photosynthesis under lower temperatures was generally more efficiently functioning than respiration, while the opposite was true for higher temperatures, where respiration typically had enhanced activity rates compared to photosynthesis. The application of the model of Blanchard et al. (1996) specifies these findings. This is in accordance to Hancke and Glud (2004), who found for microphytobenthic communities exponentially increasing respiration rates with rising temperatures but without a pronounced optimum. Higher or lower temperatures than those below the 20% percentile will best reflect thermal stress (Eggert et al., 2003).

The comparison of the calculated activation energy (*E_*a*_*) between photosynthesis and respiration of the benthic Baltic Sea diatoms studied also indicates different temperature requirements for photosynthesis and respiration. However, a closer look on the data (Table 4) shows a slightly heterogeneous response pattern, as 5 out of 8 species confirm lower temperature requirements for photosynthesis then for respiration (Karsten et al., 2014; Padfield et al., 2016; Pierangelini et al., 2019), while the remaining 3 taxa do not follow this trend. Additionally, using a very similar methodological approach, terrestrial microalgal species studied by Pierangelini et al. (2019) exhibited slightly lower activation energies for both respiratory and photosynthetic processes, thereby allowing the conclusion that metabolic rates in benthic diatoms are activated faster than those in terrestrial microalgae under increasing temperatures.

Metabolic rates of phytoplankton are increased by rising temperature (Gillooly et al., 2001; Brown et al., 2004). These metabolic rates, however, are also dependent on nutrient supply. Maranon et al. (2018) reported that nutrient limitation besides temperature is a major component that has a suppressing influence on the activation energy for metabolic processes. These authors provided an explanation in the carbon fixation mechanism, that can be maintained under lower temperatures due to the abundance and specificity of the carbon fixing enzyme RuBisCO (Maranon et al., 2018), and as already discussed above. Measurements of photosynthetic and respiratory responses as function of increasing temperature also reflect species-specific activation energies for the thermally decoupled photosynthesis and respiration. Even though both processes are not yet fully understood in benthic diatoms of the Baltic Sea, it is assumed that these different thermal requirements allow rapid acclimation to changes in fluctuating temperature conditions, which are typical for shallow water habitats (Jurasinski et al., 2018), thereby maintaining carbon transfer for efficient cell growth (Padfield et al., 2016; Pierangelini et al., 2019).

The conspicuously different temperature requirements for photosynthesis and respiration can be explained by the fact that the first process is more dependent on light-related processes (light-absorption, energy transfer etc.) than on temperature, while the second one is light-independent and thus mainly controlled by temperature (Atkin and Tjoelker, 2003). Diatom respiration consists of a set of catabolic reactions, localized in different cellular compartments and controlled by a set of specific enzymes, of which many exhibit different temperature optima. In case of the Baltic Sea species the measured temperature optima lie in the range of values typical for organisms from the temperate to warm-temperate region.

Comparing the *PE*-curve data (Figure 1) with those from the temperature experiment (Figure 2) indicate some inconsistencies between the maximum photosynthetic rates (NPP_max_) at 20°C. While in *M. moniliformis*, *Halamphora* sp. 1, *N. phyllepta*, and *N. pusilla* both experimental treatments led to almost identical NPP_max_ values, in *A. octonarius* and *N. perminuta* NPP_max_ rates were 1.7–2.5 fold higher in the *PE*-curve experiment. In contrast in *Halamphora* sp. 2 and *N. dubiiformis* NPP_max_ values were 2.2–3.5 fold higher in the temperature experiment. These data indicate that the temperature effect on photosynthesis and respiration is species-dependent, and that the diatom strains respond differently to the experimental design. All cultures were kept and exponentially grew at 20°C, and hence the *PE*-curve data (recorded at 20°C) reflect long-term acclimation to this temperature. In contrast the temperature experiment included rather drastic temperature changes over short time intervals, which are unusual in the natural habitat, but provide important information on the ecophysiological performance, plasticity and tolerance width of each species. The most important conclusion which can be drawn from the data presented is, that diatoms of a microphytobenthic community exhibit an array of specific temperature tolerances from stenothermal to eurythermal response patterns, which might explain the ecological success of such sedimentary phototrophic biofilms under fluctuating temperature conditions in shallow coastal water habitats all over the world (Cahoon, 1999). The underlying mechanisms are unstudied, but might be explained by species-specific temperature-induced difference in biochemical responses as discussed above.

### The Effect of Hütelmoor Water Components on Growth

Coastal areas are under high environmental pressure due to natural dynamics from the terrestrial and the marine sites as well as due to anthropogenic effects such as construction of coastal protection measures and global change. Some of these areas, such as the Hütelmoor, are now undergoing restoration forcing an exchange between land and sea processes and fluxes (Jurasinski et al., 2018).

Export of nutrients and organic compounds from the coastal peatland into the adjacent shallow water zone can be expected, which might stimulate growth of benthic diatoms and other microorganisms. Indeed, the Hütelmoor water was strongly enriched with all essential nutrients compared to Baltic Sea water. The growth assays with *N. dubiiformis* and *A. octonarius* experimentally prove for the first time, that peatland water has a stimulating effect on growth in the light, which can be explained by the higher nutrient concentrations in the Hütelmoor water media. In addition, while *N. dubiiformis* did not grow in the dark, growth of *A. octonarius* occurred in all media when kept in the dark. However, the significantly higher growth of *A. octonarius* in Hütelmoor compared to Baltic Sea media in the dark, cannot be explained by higher nutrient values. Measurements of the organic carbon revealed about 20 times higher organic content in Hütelmoor based media pointing to organic compounds which might support heterotrophic growth under dark conditions. Heterotrophy in diatoms is a well described metabolic capability, for example, in diatoms living inside and underneath the sea ice in the polar regions, or when buried in sediments (Tuchman et al., 2006; Morales-Sánchez et al., 2014). A wide range of carbon sources for heterotrophic metabolism in phototrophs have been identified including acetate, lactate and glucose (Tuchman et al., 2006; Wang et al., 2012).

Heterotrophic capabilities seem to be an ecological advantage for benthic diatoms in shallow water zones, because hydrodynamic and meteorological conditions at the southern Baltic Sea coast are characterized by strong wind, waves and currents causing regular sediment resuspension. Under these rather instable conditions benthic diatoms, which are often strongly attached to sand grains (K. Kuriyama, personal communication) get regularly buried, and the switch from a phototrophic to a heterotrophic mode might contribute to survival. Polar benthic diatoms can survive several months of darkness by utilization of internal storage compounds, such as the lipid compound triacylglycerol (Schaub et al., 2017). To our knowledge, no other study indicating stimulating effect of coastal peatland water on growth of nearshore benthic diatoms has been published. Therefore, more experimental investigations are needed to better understand biogeochemical processes and their ecological consequences across the land-sea interface.

## Conclusion

Overall, the studied benthic diatom strains exhibited a high photo-physiological plasticity under increasing photon fluence rates (0–1200 μmol photons m^–2^ s^–1^), along with broad maximum net primary production rates and lack of pronounced photoinhibition. In addition, these diatoms can be characterized as eurythermal organisms. All these ecophysiological data indicate a high tolerance of benthic diatoms against the fluctuating environmental conditions in the shallow water coastal zone of the southern Baltic Sea. In addition, first experimental results point to heterotrophic capabilities, at least in some strains, which is fueled by organic coastal peatland compounds. These data document the importance of follow-up studies to better characterize and understand exchange processes and their ecological consequences along terrestrial-marine gradients.

## Data Availability

Upon acceptance of the article all raw data will be published on PANGAEA and National Center for Biotechnology Information (NCBI) website.

## Author Contributions

AG, SG-P, and UK developed the idea and elaborated the concept. LP, AG, SG-P, VS, and KK provided experimental or taxonomic data. All authors organized and conducted the data analyses. LP, AG, and SG-P wrote the first draft of the manuscript, which was commented and edited by all other authors.

## Conflict of Interest Statement

The authors declare that the research was conducted in the absence of any commercial or financial relationships that could be construed as a potential conflict of interest.

## Footnotes

1 https://www.baltic-transcoast.uni-rostock.de

2 www.algaebase.org

## References

[B1] Abarca N. Jahn R. Zimmermann J. Enke N. (2014). Does the cosmopolitan diatom gomphonema parvulum (Kützing) Kützing have a biogeography? *PLoS One* 9:e086885. 10.1371/journal.pone.0086885 24489799 PMC3906111

[B2] Adir N. Zer H. Shochat S. Ohad I. (2003). Photoinhibition - a historical perspective. *Photosynth. Res.* 76 343–370. 16228592 10.1023/A:1024969518145

[B3] Admiraal W. (1984). The ecology of estuarine sediment inhabiting diatoms. *Prog. Phycol. Res.* 3 269–322.

[B4] Allakhverdiev S. I. Kreslavski V. D. Klimov V. V. Los D. A. Carpentier R. Mohanty P. (2008). Heat stress: an overview of molecular responses in photosynthesis. *Photosynth. Res.* 98 541–550. 10.1007/s11120-008-9331-0 18649006

[B5] Allen A. P. Gillooly J. F. Brown J. H. (2005). Linking the global carbon cycle to individual metabolism. *Funct. Ecol.* 19 202–213. 10.1021/acs.est.7b00689 28602082 PMC5871744

[B6] Apel K. Hirt H. (2004). Reactive oxygen species: metabolism, oxidative stress, and signal transduction. *Ann. Rev. Plant Biol.* 55 373–399. 15377225 10.1146/annurev.arplant.55.031903.141701

[B7] Armbrust E. V. (2009). The life of diatoms in the world’s oceans. *Nature* 459 185–192. 19444204 10.1038/nature08057

[B8] Aslam S. N. Strauss J. Thomas D. N. Mock T. Underwood G. J. C. (2018). Identifying metabolic pathways for production of extracellular polymeric substances by the diatom *Fragilariopsis cylindrus* inhabiting sea ice. *ISME J.* 12 1237–1251. 10.1038/s41396-017-0039-z 29348581 PMC5932028

[B9] Atkin O. K. Tjoelker M. G. (2003). Thermal acclimation and the dynamic response of plant respiration to temperature. *Trends Plant Sci.* 8 343–351. 12878019 10.1016/S1360-1385(03)00136-5

[B10] Bailleul B. Rogato A. de Martino A. Coesel S. Cardol P. Bowler C. (2010). An atypical member of the light-harvesting complex stress-related protein family modulates diatom responses to light. *Proc. Natl. Acad. Sci. U.S.A.* 42 18214–18219. 10.1073/pnas.1007703107 20921421 PMC2964204

[B11] Barrett R. D. H. Schluter D. (2008). Adaptation from standing genetic variations. *Ecol. Evol.* 23 38–44. 10.1016/j.tree.2007.09.008 18006185

[B12] Beninger P. G. Cuadrado D. Van de Koppel J. (2019). Sedimentary and biological patterns on mudflats. *Acat. Ecol. Ser.* 7 185–211. 10.1086/652991 20497053

[B13] Bischof K. Hanelt D. Tüg H. Karsten U. Brouwer P. E. M. Wiencke C. (1998). Acclimation of brown algal photosynthesis to ultraviolet radiation in arctic coastal waters (Spitzbergen, Norway). *Polar Biol.* 20 388–395.

[B14] Blanchard G. F. Guarini J. M. Richard P. Gros P. Mornet F. (1996). Quantifying the short-term temperature effect on light-saturated photosynthesis of intertidal microphytobenthos. *Mar. Ecol. Prog. Ser.* 134 309–313.

[B15] Blommaert L. Lavaud J. Vyverman W. Sabbe K. (2018). Behavioural versus physiological photoprotection in epipelic and epipsammic benthic diatoms. *Eur. J. Phycol.* 53 146–155.

[B16] Bohórquez J. McGenity T. J. Papaspyrou S. García-Robledo E. Corzo A. Underwood G. J. C. (2018). Different types of diatom-derived extracellular polymeric substances drive changes in heterotrophic bacterial communities from intertidal sediments. *Front. Microbiol.* 8:245. 10.3389/fmicb.2017.00245 28289404 PMC5326797

[B17] Bojko M. Olchawa-Pajor M. Goss R. Schaller-Laudel S. Strzalka K. Latowski D. (2019). Diadinoxanthin de-epoxidation as important factor in the short-term stabilization of diatom photosynthetic membranes exposed to different temperatures. *Plant Cell Environ.* 42 1270–1286. 10.1111/pce.13469 30362127

[B18] Brown J. H. Gillooly J. F. Allen A. P. Savage V. M. West G. B. (2004). Towards a metabolic theory of ecology. *Ecology* 85 1771–1789.

[B19] Cahoon L. B. (1999). The role of benthic microalgae in neritic ecosystems. *Oceanogr. Mar. Biol. Ann. Rev.* 37 47–86.

[B20] Choudhury E. K. Rivero R. M. Blumwald E. Mittler R. (2017). Reactive oxygen species, abiotic stress and stress combination. *Plant J.* 90 856–867. 27801967 10.1111/tpj.13299

[B21] Cohn S. A. Disparti N. C. (1994). Environmental factors influencing diatom cell motility. *J. Phycol.* 30 818–828.

[B22] Colijn F. Buurt G. (1975). Influence of light and temperature on the photosynthetic rate of marine benthic diatoms. *Mar. Biol.* 31 209–214.

[B23] Colijn F. De Jonge V. N. (1984). Primary production of microphytobenthos in the ems-dollard estuary. *Mar. Ecol. Prog. Ser.* 14 185–196.

[B24] Crafts-Brandner S. J. Salvucci S. J. (2000). Rubisco activase constrains the photosynthetic potential of leaves at high temperature and CO2. *Proc. Natl. Acad. Sci. U.S.A.* 97 13430–13435. 11069297 10.1073/pnas.230451497 PMC27241

[B25] De Brouwer J. F. C. Wolfstein K. Ruddy G. K. Jones T. E. R. Stal L. J. (2005). Biogenic stabilization of intertidal sediments: the importance of extracellular polymeric substances produced by benthic diatoms. *Microb. Ecol.* 49 501–512. 16052376 10.1007/s00248-004-0020-z

[B26] Delwiche C. F. (2007). “Algae in the warp and weave of life: bound by plastids,” in *Unravelling the Algae - the Past, Present, and Future of Algal Systematics*, eds Brodie J. Lewis J. (Boca Raton, FL: CRC Press), 7–20.

[B27] Eggert A. Burger E. M. Breeman A. M. (2003). Ecotypic differentiation in thermal traits in the tropical to warm-temperate green macrophyte *Valonia utricularis*. *Botan. Mar.* 48 69–81.

[B28] Epping E. H. G. Jørgensen B. B. (1996). Light enhanced oxygen respiration in benthic phototrophic communities. *Mar. Ecol. Progr. Ser.* 139 193–203.

[B29] Falkowski P. G. Barber R. T. Smetacek V. (1998). Biogeochemical controls and feedbacks on ocean primary production. *Science* 281 200–206. 9660741 10.1126/science.281.5374.200

[B30] Falkowski P. G. Owens T. G. (1980). Light-shade adaption; two strategies in marine phytoplankton. *Plant Physiol.* 66 592–595. 16661484 10.1104/pp.66.4.592 PMC440685

[B31] Falkowski P. G. Raven J. A. (1997). *Aquatic Photosynthesis.* Oxford: Blackwell, 375.

[B32] Gillooly J. F. Brown J. H. West G. B. Savage V. M. Charnoy E. L. (2001). Effects of size and temperature on metabolic rate. *Science* 293 2248–2251. 11567137 10.1126/science.1061967

[B33] Goss R. Lepetit B. (2015). Biodiversity of NPQ. *J. Plant Physiol.* 172 13–32. 10.1016/j.jplph.2014.03.004 24854581

[B34] Goto N. Mitamura O. Terai H. (2001). Biodegradation of photosynthetically produced extracellular organic carbon from intertidal benthic algae. *J. Exp. Mar. Biol. Ecol.* 257 73–86. 11165300 10.1016/s0022-0981(00)00329-4

[B35] Grützmacher F. (2009). *Moore – Lebensräume mit Hoher Bedeutung für Natur- und Klimaschutz.* Berlin: Naturschutzbund Deutschland.

[B36] Guillard R. R. L. (1975). *Culture of Phytoplankton for Feeding Marine Invertebrates. Culture of Marine Invertebrate Animals.* New York, NY: Plenum Press, 26–60.

[B37] Guillard R. R. L. Ryther J. H. (1962). Studies or marine planktonic diatoms. I. cyclotella nana hustedt and detonula confervacea cleve. *Can. J. Microbiol.* 8 229–239. 13902807 10.1139/m62-029

[B38] Hancke K. Glud R. N. (2004). Temperature effects on respiration and photosynthesis in three diatom-dominated benthic communities. *Aqu. Microb. Ecol.* 37 265–281.

[B39] Hanelt D. Wiencke C. Karsten U. Nultsch W. (1997). Photoinhibition and recovery after high light stress in different developmental and life history stages of *Laminaria saccharina* (Phaeophyta). *J. Phycol.* 33 387–395.

[B40] Hillebrand H. Sommer U. (1997). Response of epilithic microphytobenthos of the western Baltic Sea to *in situ* experiments with nutrient enrichment. *Mar. Ecol. Prog. Ser.* 160 35–46.

[B41] Hopkins T. J. (1964). A study of the diatoms of the Ouse Estuary, Sussex I. the movement of the mudflat diatoms in response to some chemical and physical changes. *J. Mar. Biol. Assoc. U.K.* 43 653–663.

[B42] Jurasinski G. Janssen M. Voss M. Böttcher M. E. Brede M. Burchard H. (2018). Understanding the coastal ecocline: assessing sea-land-interactions at non-tidal, low-lying coasts through interdisciplinary research. *Front. Mar. Sci.* 5:342 10.3389/fmars.2018.00342

[B43] Karsten U. Herburger K. Holzinger A. (2014). Dehydration, temperature, and light tolerance in members of the aeroterrestrial green algal genus *Interfilum* (*Streptophyta*) from biogeographically different temperate soils. *J. Phycol.* 50 804–816. 10.1111/jpy.12210 25810561 PMC4370238

[B44] Karsten U. Lütz C. Holzinger A. (2010). Ecophysiological performance of the aeroterrestrial green alga *Klebsormidium crenulatum* (*Charophyceae*, *Streptophyta*) isolated from an alpine soil crust with an emphasis on desiccation stress. *Phycologia* 46 1187–1197.

[B45] Kitano M. Matsukawa R. Karube I. (1997). Changes in eicosapentaenoic acid content of *Navicula* saprophila, *Rhodomonas salina* and *Nitzschia* sp. under mixotrophic conditions. *J. Appl. Phycol.* 9 559–563.

[B46] Krammer K. Lange-Bertalot H. (2007). *Süßwasserflora von Mitteleuropa; Bacillariophyceae; Band 2/1.* Berlin: Springer Spektrum.

[B47] Krammer K. Lange-Bertalot H. (2010). *Süßwasserflora Von Mitteleuropa; Bacillariophyceae; Band 2/2.* Berlin: Springer Spektrum.

[B48] Lange-Bertalot H. (2000). *Iconographia Diatomologica: Annotated Diatom Micrographs.* Ruggell, FL: Gantner Verlag.

[B49] Lange-Bertalot H. (2013). *Diatomeen im Süßwasser-Benthos Von Mitteleuropa.* Oberreifenberg: Koeltz Scientific Books.

[B50] Launeau P. Méléder V. Verpoorter C. Barillé L. Kazemipour-Ricci F. Giraud M. (2018). Microphytobenthos biomass and diversity mapping at different spatial scales wither hyperspectral optical model. *Remote Sens.* 10:716.

[B51] Lavaud J. Strzepe R. F. Kroth P. G. (2007). Photoprotection capacity differs among diatoms: Possible consequences on the spatial distribution of diatoms related to fluctuations in the underwater light climate. *Limnol. Oceanogr.* 52 1188–1194.

[B52] López-Urrutia A. San Martin E. Harris R. P. Irigoien X. (2006). Scaling the metabolic balance of the oceans. *Proc. Natl. Acad. Sci. U.S.A.* 103 8739–8744. 16731624 10.1073/pnas.0601137103 PMC1482648

[B53] Maranon E. Lorenzo M. Cermeno P. Mourino-Carballido B. (2018). Nutrient limitation suppresses the temperature dependence of phytoplankton metabolic rates. *ISME J.* 12 1836–1845. 10.1038/s41396-018-0105-1 29695860 PMC6018665

[B54] Meyercordt J. Gerbersdorf S. Meyer-Reil L.-A. (1999). Significance of pelagic and benthic primary production in two shallow coastal lagoons of different degrees of eutrophication in the southern Baltic Sea. *Aquat. Microb. Ecol.* 20 273–284. 10.3354/ame020273

[B55] Meyercordt J. Meyer-Reil L.-A. (1999). Primary production of benthic microalgae in two shallow coastal lagoons of different trophic status in the southern Baltic Sea. *Mar. Ecol. Prog. Ser.* 178 179–191. 10.3354/meps178179

[B56] Miegel K. Graeff T. Franck C. Salzmann T. Bronstert A. Walther M. (2017). Auswirkungen des Sturmhochwassers der Ostsee am 4./5. Januar 2017 auf das renaturierte niedermoor ,,hütelmoor und heiligensee“ an der deutschen Ostseeküste. *Hydrol. Wasserbewirtsch.* 61 232–243.

[B57] Miller D. C. Geider R. J. MacIntyre H. L. (1996). Microphytobenthos: The ecological role of the ‘secret garden’ of unvegetated, shallow-water marine habitats. II. role in sediment stability and shallow-water food webs. *Estuaries* 19 202–212.

[B58] Mohanty P. Allakhverdiev S. I. Murata N. (2007). Application of low temperature during photoinhibition allows characterization of individual steps in photodamage and repair of photosystem II. *Photosynth. Res.* 94 217–234. 17554634 10.1007/s11120-007-9184-y

[B59] Morales-Sánchez D. Martiniez-Rodriguez O. A. Kyndt J. Martinez A. (2014). Heterotrophic growth of microalgae: metabolic aspects. *World J. Microbiol. Biotechnol.* 31 1–9. 25388473 10.1007/s11274-014-1773-2

[B60] Morel F. M. M. Price N. M. (2003). The biochemical cycle of trace elements in the oceans. *Science* 300 944–947. 12738853 10.1126/science.1083545

[B61] Mortain-Bertrand A. Descolas-Gros C. Jupin H. (1988). Growth, photosynthesis and carbon metabolism in the temperate marine diatom *Skeletonema costatum* adapted to low temperature and low photon-flux density. *Mar. Biol.* 100 135–141.

[B62] Mouget J. L. Perkins R. G. Consalvey M. Lefebvre S. (2008). Migration or photoacclimation to prevent photoinhibition and UV-B damage in marine microphytobenthic communities. *Aquat. Microb. Ecol.* 55 223–232.

[B63] Murata N. Takahashi S. Nishiyama Y. Allakhverdiev S. I. (2007). Photoinhibition of photosystem II under environmental stress. *Biochim. Biophys. Acta* 1767 414–421. 17207454 10.1016/j.bbabio.2006.11.019

[B64] Nymark M. Valle K. C. Brembu T. Hancke K. Winge P. Andresen K. (2009). An integrated analysis of molecular acclimation to high light in the marine diatom *Phaeodactylum tricornutum*. *PLoS One* 4:e007743. 10.1371/journal.pone.0007743 19888450 PMC2766053

[B65] Padfield D. Yvon-Durocher G. Buckling A. Jennings S. Yvon-Doucher G. (2016). Rapid evolution of metabolic traits explains thermal adaptation in phytoplankton. *Ecol. Lett.* 9 133–142. 10.1111/ele.12545 26610058 PMC4991271

[B66] Perkins R. G. Lavaud J. Serôdio J. Mouget J. L. Cartaxana P. Rosa P. (2010). Vertical cell movement is a primary response of intertidal benthic biofilms to increasing light dose. *Mar. Ecol. Prog. Ser.* 416 93–103.

[B67] Pierangelini M. Glaser K. Mikhailyuk T. Karsten U. Holzinger A. (2019). Light and dehydration but not temperature drive photosynthetic adaptations of basal streptophytes (*Hormidiella*, *Streptosarcina* and *Streptofilum*) living in terrestrial habitats. *Microb. Ecol.* 77 380–393. 10.1007/s00248-018-1225-x 29974184 PMC6394494

[B68] Raven J. A. Geider R. J. (1988). Temperature and algal growth. *New Phytol.* 110 441–461.

[B69] Reusch T. B. H. Dierking J. Andersson H. C. Bonsdorff E. Carstensen J. Casini M. (2018). The Baltic Sea as a time machine for the future coastal ocean. *Sci. Adv.* 4 1–16. 10.1126/sciadv.aar8195 29750199 PMC5942908

[B70] Risgaard-Petersen N. Rysgaard S. Nielsen L. P. Revsbech N. P. (1994). Diurnal variation of denitrification and nitrification in sediments colonized by benthic microphytes. *Limnol. Oceanogr.* 39 573–579.

[B71] Rönnberg C. Bonsdorff E. (2004). Baltic Sea eutrophication: area-specific ecological consequences. *Hydrobiologia* 514 227–241.

[B72] Round F. E. (1971). Benthic marine diatoms. *Oceanogr. Mar. Biol. Ann. Rev.* 9 83–139.

[B73] Sackett O. Petroul K. Reedy B. Hill R. Doblin M. Beardall J. (2016). Snapshot prediction of carbon productivity, carbon and protein content in a Southern Ocean diatom using FTIR spectroscopy. *ISME J.* 10 416–426. 10.1038/ismej.2015.123 26230047 PMC4737933

[B74] Sagert S. Schubert H. (1999). Unterwasserlichtklima der darss-zingster-boddenkette. *Rostock Meeresbiol. Beitr.* 7 135–155.

[B75] Saks N. M. (1983). Primary production and heterotrophy of a pennate and a centric salt marsh diatom. *Mar. Biol.* 76 241–246.

[B76] Saravanan V. Godhe A. (2010). Genetic heterogeneity and physiological variation among seasonally separated clones of *Skeletonema marinoi* (*Bacillariophyceae*) in the Gullmar Fjord. Sweden. *Eur. J. Phycol.* 45 177–190.

[B77] Schaub I. Wagner H. Graeve M. Karsten U. (2017). Effects of prolonged darkness and temperature on the lipid metabolism in the benthic diatom *Navicula perminuta* from the Arctic Adventfjorden. Svalbard. *Polar Biol.* 40 1425–1439.

[B78] Scholz B. Liebezeit G. (2012). Growth responses of 25 benthic marine Wadden Sea diatoms isolated from the Solthörn tidal flat (southern North Sea) in relation to varying culture conditions. *Diatom Res.* 27 65–73.

[B79] Serodio J. Vieira S. Cruz S. Coelho H. (2006). Rapid light-response curves of chlorophyll fluorescence in microalgae: relationship to steady-state light curves and non-photochemical quenching in benthic diatom-dominated assemblages. *Photosynth. Res.* 90 29–43. 17111236 10.1007/s11120-006-9105-5

[B80] Shniukova E. I. Zolotareva E. K. (2015). Diatom exopolysaccharides: a review. *Int. J. Algae* 17 50–67.

[B81] Snoeijs P. Potapova M. (1995). *Intercalibration and Distribution of Diatom Species in the Baltic Sea.* Uppsala: Opulus Press, 3.

[B82] Snoeijs P. Vilbaste S. (1994). *Intercalibration and Distribution of Diatom Species in the Baltic Sea.* Uppsala: Opulus Press, 2.

[B83] Stachura-Suchoples K. Enke N. Schlie C. Schaub I. Karsten U. Jahn R. (2016). Contribution towards a morphological and molecular taxonomic reference library of benthic marine diatoms from two arctic fjords on Svalbard (Norway). *Polar Biol.* 39 1933–1956.

[B84] Sundbäck K. Miles A. (2002). Role of microphytobenthos and denitrification for nutrient turnover in embayments with floating macroalgal mats: a spring situation. *Aquat. Microb. Ecol.* 30 91–101.

[B85] Tcherkez G. G. B. Farquhar G. D. Andrews J. T. (2006). Despite slow catalysis and confused substrate specificity, all ribulose bisphosphate carboxylases may be nearly perfectly optimized. *Proc. Natl. Acad. Sci. U.S.A.* 103 7246–7251. 16641091 10.1073/pnas.0600605103 PMC1464328

[B86] Throndsen J. Sournia A. (1978). *Preservation and Storage. Phytoplankton Manual.* Paris: UNESCO, 69–74.

[B87] Tuchman N. Schollett M. Steven T. Geddes P. (2006). Differential heterotrophic utilization of organic compounds by diatoms and bacteria under light and dark conditions. *Hydrobiologia* 1 38–43.

[B88] Turesson G. (1922). The species and the variety as ecological units. *Hereditas* 3 100–113.

[B89] Utermöhl H. (1958). The improvement of quantitative phytoplankton methodology. International Association of Theoretical and Applied Limnology. *Comm. Limnol. Methods* 9 1–39.

[B90] Villanova V. Fortunato A. E. Singh D. Bo D. D. Conte M. Obata T. (2017). Investigating mixotrophic metabolism in the model diatom *Phaeodactylum tricornutum*. *Philos. Trans. R. Soc. B* 372:20160404. 10.1098/rstb.2016.0404 28717014 PMC5516113

[B91] Wagner H. Jakob T. Wilhelm C. (2006). Balancing the energy flow from captured light to biomass under fluctuating light conditions. *New Phytol.* 169 95–108. 16390422 10.1111/j.1469-8137.2005.01550.x

[B92] Walsby A. E. (1997). Numerical integration of phytoplankton photosynthesis through time and depth in a water column. *New Phytol.* 136 189–209.

[B93] Wang H. Fu R. Pei G. (2012). A study on lipid production of the mixotrophic microalgae *Phaeodactylum tricornutum* on various carbon sources. *Afr. J. Microbiol. Res.* 6 1041–1047.

[B94] Wasmund N. Uhlig S. (2003). Phytoplankton trends in the Baltic Sea. *ICES J. Mar. Sci.* 60 177–186.

[B95] Wilhelm C. Büchel C. Fisahn J. Goss R. Jakob T. La Roche J. (2006). The regulation of carbon and nutrient assimilation in diatoms is significantly different from green algae. *Protist* 157 91–124. 16621693 10.1016/j.protis.2006.02.003

[B96] Woelfel J. Schoknecht A. Schaub I. Enke N. Schuhmann R. Karsten U. (2014). Growth and photosynthesis characteristics of three benthic diatoms from the brackish southern Baltic Sea in relation to varying environmental conditions. *Phycologia* 53 639–651.

[B97] Wolfstein K. Stal L. J. (2002). Production of extracellular polymeric substances (EPS) by benthic diatoms: effect of irradiance and temperature. *Mar. Ecol. Progr. Ser.* 236 13–22.

[B98] Wulff A. Sundbäck K. Nilsson C. Carlson L. Jönsson B. (1997). Effect of sediment load on the microbenthic community of a shallow-water sandy sediment. *Estuaries* 20 547–558. 10.2307/1352613

[B99] Young J. N. Goldman J. A. Kranz S. A. Torell P. D. Morel F. M. (2014). Slow carboxylation of Rubisco constrains the maximum rate of carbon fixation during Antarctic phytoplankton blooms. *New Phytol.* 205 172–181. 10.1111/nph.13021 25283055

[B100] Zimmermann J. Jahn R. Gemeinholzer B. (2011). Barcoding diatoms: evaluation of the V4 subregion on the 18S rRNA gene, including new primers and protocols. *Organ. Diver. Evol.* 11 173–192.

